# 

**Published:** 1921-04-16

**Authors:** 


April 16, 1921] [Price Twopence

### The Hospital

The Workers' Newspaper of Administrative Medicine and Institutional Life, Administration, National Insurance and Health.

### CONTENTS

**A C 3 "Bogey"** ... ... ... ... ... ... 39

**A General Effort** ... ... ... ... ... ... 39

**The Edinburgh Tuberculosis Decision** ... ... 40

**Points from Annual Reports** ... ... ... ... 40

**Notes of the Week** ... ... ... ... ... 41
The Legality of Municipal Hospitals—The Approved Societies—The New Anæsthetic—Medicine in Brussels—Research in Tropical Disease—Filtering of Tubercular Cases—Warranties Under the Food and Drugs Acts—A Great Floating Hospital—New Secretary of Westminster Hospital—The Cultivation of Sunday Schools—Treatment of Limbless in Wales—Supervision of Blind Charities—Hospital Life in Cork—Record Saturday Fund Collection.

**The Cause of Industrial Cancer** ... ... ... 43
A First Publication by the International Labour Office.

**The Perfect Foot** ... ... ... ... ... ... 44

Some Orthopædic Principles Explained.

**Hospital Neighbourhoods** ... ... ... ... 45

II. The City Road

**The Public Well-Being** ... ... ... ... ... 47

Health in the Schools—The Rat Pest—Lectures to Patients.

**King Edward's Hospital Fund for London** ... ... 49

The Events of a Critical Period.

**Passing Events and Topics** ... ... ... ... 50

**The Editor's Letter-Box** .. ... ... ... ... 51
On Looking the Age of Consent—Village and Country Town Concerts—Should Nurses Strike?

**Parliament and the Professions** ... ... ... 51

**Annual Meetings of Hospitals** ... ... ... ... 52

**The Matrons' and Sisters' Department** ... ... 53

The Pay of the Nurse.—VI. The Lack of Superannuation.

**Round the Hospitals** ... ... ... ... 53

**Matrons' and Sisters' Appointments** ... ... 55

**The College of Nursing (Limited by Guarantee)** 56
What the Local Centres are Doing.

**General Nursing Council for Scotland** ... ... 56

